# Elevated Risk of Suicidal Ideation in HIV-Positive Persons

**DOI:** 10.1155/2015/609172

**Published:** 2015-09-29

**Authors:** L. Schlebusch, R. D. Govender

**Affiliations:** ^1^Department of Behavioural Medicine, Nelson R Mandela School of Medicine, University of KwaZulu-Natal, Durban, South Africa; ^2^Department of Family Medicine, Nelson R Mandela School of Medicine, University of KwaZulu-Natal, Durban, South Africa

## Abstract

Globally, suicide and HIV/AIDS remain two of the greatest healthcare issues, particularly in low- and middle-income countries. Several studies have observed a relationship between suicidal behaviour and HIV/AIDS.* Materials and Methods*. The main objective of this research was to determine the prevalence of elevated risk of suicidal ideation in HIV-positive persons immediately following voluntary HIV counselling and testing (VCT). The study sample consisted of adult volunteers attending the VCT clinic at a university-affiliated, general state hospital. Participants completed a sociodemographic questionnaire, Beck's Hopeless Scale, and Beck's Depression Inventory.* Results*. A significantly elevated risk of suicidal ideation was found in 83.1% of the patients who tested seropositive. Despite a wide age range in the cohort studied, the majority of patients with suicidal ideation were males in the younger age group (age < 30 years), consistent with the age-related spread of the disease and an increase in suicidal behaviour in younger people. Relevant associated variables are discussed.* Conclusion*. The results serve as important markers that could alert healthcare professionals to underlying suicide risks in HIV-positive patients. It is recommended that screening for elevated risk of suicidal ideation and prevention of suicidal behaviour should form a routine aspect of comprehensive patient care at VCT clinics.

## 1. **Introduction**

Suicidal behaviour and HIV/AIDS continue to present major public health challenges especially in low- and middle-income countries [1–4]. Although there are wide variations between and within countries and subregions, research has shown that under certain circumstances HIV/AIDS patients can be at high risk for suicidal behaviour [5–9]. According to the World Health Organization (WHO), in the past few decades, global suicide rates have increased by 60% with nearly one million people worldwide dying each year as a consequence of suicide which is likely to increase to predicted suicide mortality rates of 1.53 million per annum by the year 2020 [1]. Likewise, global HIV/AIDS trends show that the number of people living with HIV/AIDS rose from around 8 million in 1990 to 34 million by the end of 2010, with more than 25 million people dying from it since it was first diagnosed in 1981 with sub-Saharan Africa carrying the greatest burden of the epidemic and Southern Africa being the most heavily affected (up to 68% of all HIV-positive people) [7, 10, 11]. South Africa has been estimated to have one of the world's largest populations of people living with HIV/AIDS (5.7 million) [4, 10, 12]. Durban is a large harbour city with one of the busiest ports in Africa and is situated on the eastern seaboard of the country in the Province of KwaZulu-Natal (where the research reported in the present paper was conducted). Some 60% of the province's economic activity takes place in this port city where 88.6% of its inhabitants are black South Africans and 68% are of working age (16–65 years), but up to one-third or more are unemployed [13]. This geographical region bears not only one of the highest HIV-seropositive prevalence rates, but also high comparative suicidal ideation rates in the general population as found in a community survey component of a large WHO international multisite study conducted in nine cities on five continents [1, 14]. Although the results of that study showed a remarkable disparity across the sites where the research was done (illustrating how careful one must be when making national, international, and/or regional comparisons), the proportion of subjects in the general population in Durban that admitted to having had suicidal thoughts in their lifetime was 25.4%. Country wide, South African suicide rates covering different years are equally alarming with mean annual estimates ranging from 10.9 to as high as 25 per 100,000 of the population depending on sample size and where and when the research was done [13, 15–18], but indicating a general trend that suicidal behaviour is on the increase, especially among younger people [5, 15, 19]. Several non-hospital based South African studies [15] showed that between 4% and 47% of school children surveyed expressed suicidal ideation, while in a national survey in a sample of persons aged 18 years and older the estimated lifetime prevalence rate of suicidal ideation was 9.1% [20].

Similarly, research on suicidal ideation in HIV/AIDS patients has produced diverse results. Suicidal behaviour stems from a complex constellation of components and how they interact with the suicidal process varying from one individual to another and specific risk factors [2, 5, 21]. Not only is suicidal behaviour a complex process that ranges in degree of severity from thinking about killing oneself (i.e., suicidal ideation) to actually doing it [2, 5], but also in at-risk persons with HIV/AIDS the pattern and/or prevalence of suicidal behaviour may differ throughout the progression of the disease from HIV infection up to the development of full-blown AIDS and thereafter [5, 7]. HIV/AIDS carries several unique stressors [5, 7, 22]. When patients are first diagnosed to be HIV-positive, many react with disbelief and anxiety and express fear of what lies ahead. As early as 1995, a South African study showed that 17% of youth had attempted suicide because of an AIDS phobia [23]. Some studies suggest that certain patients attempt suicide within the first three months of notification of their positive status, whereas others do so within the first year [7, 24–26]. Others may become increasingly suicidal upon a deterioration of their medical condition during the advanced stages of the disease and the development of neurocognitive impairment or as a result of psychiatric complications [7, 26–29]. Higher rates of suicidal ideation in HIV-positive individuals generally have been described whereas some studies have found that some asymptomatic HIV-infected persons had higher rates than persons with AIDS or reported suicidal ideation in patients within the first week of testing for HIV [6, 9, 25, 30–32]. A study [24] of HIV-positive nonpsychiatric individuals found that two-thirds reported lifetime and one-third current suicidal ideation. In line with several studies [24–26, 29–32] from other countries that reported on the nexus between lifetime and current suicidal ideation in different HIV seropositive patient samples, a recent South African study reported high levels of suicidal ideation (27.5%) closely linked to suicide plans among HIV-positive pregnant women [33].

However, although various statistics are available on the prevalence of suicidal ideation in HIV-positive persons, there is limited research data on the elevated risk of suicidal ideation immediately following a positive HIV diagnosis at clinics offering voluntary HIV counselling and testing (VCT), especially in developing societies such as those found in South Africa. Consequently, the purpose of the present research was to assess such risk in this cohort of patients. VCT offers patients a chance to comprehend their HIV status and, depending on the results of testing, affords them the opportunity to immediately access treatment and ultimately presents scope for them to positively alter suicidal risk behaviour, if present. This paper emanates from and is based on the results of a larger study that has produced several publications [8, 9, 34–36] where the theoretical framework underpinning the research was, in part, to assess the psychological impact of a negative life event, that is, immediately after receiving a positive HIV test result and the resultant cascade of emotions, including those that trigger elevated risk of suicidal ideation.

## 2. **Methodology**

Sample recruitment consisted of 190 adult volunteer patients referred to the VCT clinic at a university-affiliated, general state hospital in the port city of Durban (mentioned earlier), South Africa, over a three-month period. One patient dropped out because of incomplete results, leaving 189 (99.5%) of the originally recruited number of whom 157 (83.1%) tested HIV-positive. They then voluntarily signed an informed consent form and received extensive pre- and posttest counselling according to individual patient requirements. The instruments used to assess the prevalence of suicidal ideation in recently diagnosed HIV-positive persons were a sociodemographic questionnaire, the Beck Hopelessness Scale (BHS) [37], and the Beck Depression Inventory (BDI) [38]. In accordance with previous research [9, 34], we used a cut-off score of 9 and above on the BHS to identify suicidal ideation in this study. For the BDI, we used the standard cut-off scores, namely, 0–9 (minimal depression), 10–18 (mild depression), 19–29 (moderate depression), and 30–63 (severe depression). Participants completed the questionnaires at 72 hours and again at 6 weeks following VCT. SPSS software (version 15.0; SPSS Inc., Chicago, Illinois, USA) was used for data analysis, along with Pearson's chi-squared test,* t*-tests, and a binary logistic regression analysis of the sociodemographic variables. The latter used a backward stepwise method with entry and exit probabilities set at 0.05 and 0.1 based on likelihood ratio tests. The sociodemographic variables were entered into the model at step one. After five steps, the final model was reported with odds ratios (ORs) and 95% confidence intervals (CIs). In addition, to ascertain the sensitivity and specificity of cut-off points on the BDI to predict suicidal ideation, receiver operating characteristic (ROC) analyses were utilised. It yielded a summary measure, which is the area under the curve (AUC), which was considered as the probability of correct prediction [34].

Ethical approval was obtained from the University of KwaZulu-Natal (UKZN) Biomedical Research and Ethics Committee and the provincial Department of Health. The study participants were advised of the study either in English or isiZulu (the major local language spoken) as requested. All research participants' rights (including that to confidentiality) were maintained and respected. In terms of data management and storage, all electronic databases were password-protected and the raw data were kept in a lockable cabinet, to which only the researchers had access. If the researcher suspected that a participant was at high risk for suicide, then the relevant referral for help was made for more intensive treatment.

## 3. **Results and Discussion**

In the 83.1% of the patients who tested HIV-positive, the risk of suicidal ideation was 20.5% at 72 hours, while 6 weeks thereafter the risk had increased to 28.8%. The findings represent an elevated suicidal ideation risk which was significantly associated with a diagnosis of a seropositive HIV status (*p* = 0.013; 95% CI 13.97–29.92). None of the HIV-negative patients displayed suicidal ideation after being informed of their seronegative status. The mean age was 33.49 years (SD = 9.449) for HIV-positive patients and 37.94 years (SD = 15.238) for HIV-negative patients, with an overall patient participant age range of 16–79 years in line with that of the working population of the region [13]. Of the total sample, 70.8% were females and 59.4% were unemployed individuals with the majority of patients (38.5%) falling within the 21–30-year age category. A significant association was found between HIV test results and age, while on the binary logistic regression analysis both age and gender were significant predictors of suicidal ideation (OR = 0.959, *p* = 060) with males having a 1.8 times higher risk of suicidal ideation than females (*p* = 0.025), along with a lower level of education, more significant socioeconomic pressures, and exposure to the adverse influences of traditional beliefs. These findings are consistent with other researches [3, 5, 7, 9, 13, 15, 39] that have shown an association between female vulnerability and the roles of adverse socioeconomic correlates and unemployment and low educational status and HIV infections and suicidal behaviour in sub-Saharan Africa. In terms of traditional beliefs, although researchers [2] have commented on the potential importance of cultural variables on suicidality, it has been emphasised [5] that in developing societies more research is required on the influential role of traditional beliefs in shaping responses to health behaviour and health messages. For example, some studies [7] have found that in certain beliefs HIV/AIDS is sometimes conceptualised as a mystical force resulting in negative social discourse around it, and consequently with a diminished resistance to live with it being suggestive of a “social death” experience [7]. Despite the fact that a wide age range was represented in the cohort studied, the majority of seropositive patients with suicidal ideation fell within the younger age group (age < 30 years), consistent with the age-related spread of the disease and the increase in suicidal behaviour in younger people [4, 13, 15, 40]. There have been recent South African reports of HIV-related “treatment fatigue” and suicide in teenagers [40].

Apart from a host of other stressors [7, 22], it is well known that people living with HIV/AIDS can experience stigma and discrimination in certain societies [5, 7, 41, 42]. At an individual level, stigma undermines a person's identity and capacity to cope with the disease, limits the possibility of disclosure, and hinders an eventual alteration of risk behaviour. Closely linked to stigmatisation are discrimination and fear of the disclosure of an individual's HIV/AIDS status. Blame is often assigned to partners for infecting them and can result in violence, dysfunctional households, and suicidal behaviour [5, 7]. These types of reactions inevitably can have the unfavourable outcome of rendering the epidemic “invisible” and forcing people who have contracted HIV/AIDS or those who are associated with it “to go into hiding,” thereby contributing to feelings of hopelessness and depression [5, 7]. Furthermore, the manner in which each person experiences and copes with the disease can be reflected in their decision of whether or not to disclose their serostatus and their right to personal privacy and dignity. In this regard, of the HIV-positive participants in the present study, 20.5% had an elevated hopelessness score on the BHS at 72 hours after receiving their diagnosis, and 28.8% had an elevated BHS score at 6 weeks. The findings demonstrated a significant increase in feelings of hopelessness over 6 weeks. Likewise, of the HIV-positive participants, 82.8% were depressed according to their BDI scores at 72 hours after receiving their diagnosis, while 78.2% had a score within the depressed range at 6 weeks. Importantly, many international and South African studies [2, 5] have shown that both hopelessness and depression correlate with an increased risk in suicidal behaviour, which according to our findings also apply to HIV-positive patients seen at a VCT clinic. Item 9 of BDI is represented by options of thoughts of killing oneself, to carrying or not carrying them out. A significant association was found between the responses to this item and hopelessness as defined by the BHS cut-off scores, with *p* values of 0.036 and 0.008 at 72 hours and at 6 weeks, respectively, further confirming elevated suicidal ideation in the HIV-positive patients studied. In addition, the AUC of the ROC curve of the BDI results used to predict suicidal ideation was 0.757 (*p* < 0.001) at 72 hours and 0.788 (*p* < 0.001) at 6 weeks, indicating that the relevant BDI item was a good predictor of suicidal ideation. These results are supported by previous studies that found a correlation between hopelessness, depression, and suicidal ideation [5, 38, 43, 44].

Our findings have been invaluable in establishing baseline data on elevated suicidal risk in HIV-positive persons in order to implement appropriate suicide preventive intervention immediately following a positive HIV diagnosis. We have shown that this would be the most opportune time to administer such an intervention, that is, particularly within the first 72 hours after conveyance of a positive HIV diagnosis [36]. In our view, these initial hours can be considered as the “golden hours of suicide prevention” when diagnosing HIV positivity, since patients who are often lost to follow-up would still have the benefit of suicide intervention, particularly within the context of a developing society.

## 4. **Conclusions**

The relationship between HIV/AIDS and elevated risk of suicidal ideation has, in the past, been underresearched in developing countries. Despite the diverse findings about correlations between suicidal behaviour and HIV/AIDS, there is compelling evidence to justify screening for risk of elevated suicidal ideation and subsequent suicide risk and intervening as early as possible [36, 45, 46], especially following notification of a positive HIV test result immediately after HIV counselling and testing. Suicide risk in other potentially life-threatening medical conditions can be complicated and has been explored extensively [4], for example, in cancer [47]. In the case of HIV/AIDS, many studies in different countries [5, 48] have shown that suicidality appears to be prevalent in some individuals but that it tends to vary according to the stage of the disease. Nevertheless, it has been shown that screening for suicide, even among high-risk populations, ultimately does translate into preventing suicides and that effective suicide prevention includes early recognition and assessment of risk, immediate response, resource referrals, and follow-up management and treatment of at-risk individuals [2, 5, 35, 36].

In the resource-limited context of developing societies, suicide risk assessment and interventions at VCT clinics are hampered by a shortage of adequately trained healthcare professionals, suicide risk screening in general, and guidelines for suicide preventive interventions. HIV counsellors are typically responsible for pre- and posttest HIV counselling and psychosocial education, and they can easily be task-shifted to screen the subjects for suicide risk and provide suicide intervention strategies. It is recommended that this should become routine as part of comprehensive care at VCT clinics. At a reasonable cost and with minimal training, this could see the effective reduction of suicidal ideation and ultimate suicide.

## 5. **Study Limitations**

The sample size was not large and the overall study was confined to the immediate post-HIV test period and screening of the target population being urban-based. Certain other variables which were not measured in the study could have affected the outcome. For example, there was no information on preexisting psychiatric disorders, previous suicide attempts, or a family history of suicide, and in this regard more in-depth exploratory analyses are required.

Researchers agree that suicidal behaviour is a multifaceted and complex phenomenon. The same applies to suicidal ideation. Thus, it would have been useful to also have utilised other more comprehensive measures specifically designed to assess suicidal ideation. In addition, we had access to data at two points in time, namely, 72 hours and six weeks after diagnosis. It could have been informative to have looked at three specific groups, one which started off not suicidal, a second that started off suicidal but then had their suicidal ideations resolved, and a third group that remained suicidal throughout. It is consequently important to gain greater clarity concerning the length of time that an individual may be deemed to be at elevated risk for suicidal ideation and behaviour following a traumatic event such as being diagnosed to be HIV-positive. Longitudinal studies are, therefore, needed to determine the effectiveness of screening for elevated risk of suicidal ideation in HIV-infected persons at VCT clinics over longer follow-up time periods. This would enable differentiation between variables that are more prevalent at different stages of the disease, as well as the impact of the introduction of ARVs on suicidal ideation.
